# Sex-Specific Incubation and Body Orientation in the Black-Winged Stilt (*Himantopus himantopus*): Diel Patterns and Environmental Associations

**DOI:** 10.3390/ani16213467

**Published:** 2026-11-02

**Authors:** Monif AlRashidi, Ahmed Saleh Alali

**Affiliations:** 1 Department of Biology, Faculty of Science, University of Ha’il, Ha’il 55476, Saudi Arabia; 2 King Salman Bin Abdulaziz Royal Reserve Development Authority, Riyadh 12213, Saudi Arabia; a.alali@ksrnr.gov.sa

**Keywords:** biparental incubation, diel rhythm, wind orientation, solar orientation, shorebird, *Himantopus himantopus*

## Abstract

Biparental cooperation during incubation is particularly important in harsh environments. We quantified sex-specific incubation contributions and body orientation in Black-winged Stilts (*Himantopus himantopus*) from one-minute images at 12 nests monitored for 24 h in Saudi Arabia. Incubation partitioning, environmental associations, and sex differences in body orientation relative to the sun and wind were evaluated using nest-level resampling and generalized linear mixed models, with Holm correction for environmental tests. Male incubation share averaged 50.72% (95% CI 47.21–54.35%), without clear evidence of deviation from 50% (*p* = 0.697). Male contributions varied among two-hour periods (cyclic-shift *p* = 0.042; parametric-bootstrap likelihood-ratio *p* = 0.004), whereas the daylight–night difference was inconclusive (*p* = 0.234). No environmental predictor of incubation partitioning was supported after correction. No clear sex difference was detected in solar-relative orientation (*p* = 0.154). Although the primary four-direction (*p* = 0.015) and exploratory eight-direction (*p* = 0.038) models yielded nominal sex-related differences in wind-relative orientation, these findings were not consistently corroborated by compass-preference-adjusted and episode-onset sensitivity analyses and therefore do not establish a sex-specific wind response. Sex-by-wind-speed interactions were inconclusive (*p* = 0.071 and 0.172). Overall, near-equal daily incubation contributions can mask temporal variation in parental duties across two-hour periods.

## Simple Summary

In harsh environments, biparental incubation may be particularly important, as sharing incubation duties may help reduce the demands placed on each parent. We monitored 12 Black-winged Stilt (*Himantopus himantopus*) nests in Saudi Arabia for a complete 24 h period using one image per minute to compare male and female incubation contributions and body orientation relative to the sun and wind. All 17,280 images were rescored for attendance and body axis, with four-direction classification used in the primary models and eight-direction classification as a sensitivity analysis. Males and females contributed nearly equally over 24 h, although their contributions varied among two-hour periods. No clear sex difference in solar-relative orientation was detected. Some analyses suggested that males and females differed in their tendency to face into the wind, but this finding was not consistently supported across sensitivity analyses. These results highlight diel variation in parental care and the importance of accounting for nest-level compass preferences when interpreting directional behavior.

## 1. Introduction

Parental care occurs across diverse animal taxa, although its prevalence and form vary markedly among lineages [1,2]. Recent comparative evidence indicates that both parents participate in incubation in about 48% of avian species [3]. Incubation maintains egg temperatures within limits compatible with embryonic development [4]. This thermoregulatory role can require substantial investments of time and energy and impose physiological and reproductive costs on incubating parents [5]. These trade-offs place the division of incubation between the sexes within the broader framework of sex-specific parental investment and conflict over care [6,7].

Shared incubation may be particularly advantageous when maintaining sufficient nest attendance would be difficult for a single parent [8]. Harsh environmental conditions can alter incubation demands and the degree of parental cooperation [9,10]. Experimental manipulation of nest cover showed that greater exposure to solar radiation increased incubation effort by both sexes and the frequency of incubation changeovers, whereas experimentally increased cover produced the opposite response [10]. Recent studies further indicate that relationships between environmental conditions and incubation schedules can differ among populations and ecological contexts [11,12,13].

The temporal scale of observation is therefore important for understanding how incubation is shared. Similar daily contributions by males and females may conceal substantial differences in the timing and duration of incubation bouts, and shorebirds show considerable diversity in biparental incubation rhythms [14,15]. Sex-specific incubation schedules may reflect differences in predation risk and thermal demands. In a sexually dichromatic shorebird, more conspicuously colored males incubated predominantly at night, whereas more cryptically colored females incubated during the day [16]. Under extreme heat, however, male daytime incubation can increase, leading to a more even division of parental duties [9]. These contrasting patterns highlight the importance of examining how males and females partition incubation throughout the diel cycle.

Beyond incubation attendance, incubating adults can also adjust their body orientation in relation to wind speed and direction [17]. Orientation relative to the sun, combined with postural adjustments, can reduce radiative heat gain in adult birds [18].

The Black-winged Stilt (*Himantopus himantopus*) is a widespread shorebird [19,20] that inhabits shallow wetlands throughout the temperate and tropical Old World, excluding Australia and New Zealand [20]. Nests are typically placed on the ground in shallow depressions or scrapes, and clutches usually contain four eggs [19,20]. Both sexes participate in incubation [20,21]. At exposed nest sites, incubating adults experience solar radiation, wind, and other weather conditions [22]. Sex-specific differences in upperpart plumage allow males and females to be distinguished [20], making this species well suited for comparing incubation attendance and body orientation between the sexes.

Previous research documented changes in parental roles across reproductive stages in Black-winged Stilts [21], whereas recent work in Saudi Arabia documented high nest attendance and changes in the orientation of incubating adults relative to the position of the sun without examining sex-specific differences [22]. However, how males and females divide incubation across a complete diel cycle, whether this division varies with environmental conditions, and whether the sexes differ in body orientation relative to the sun and wind remain insufficiently understood.

Accordingly, this study aimed to investigate sex-specific variation in incubation behavior in Black-winged Stilts by (1) comparing male and female incubation contributions over 24 h, between daylight and night, and across two-hour periods; (2) examining associations between environmental conditions and the division of incubation duties; and (3) testing whether males and females differ in body orientation relative to the sun and wind, including their wind-facing responses to wind speed.

## 2. Materials and Methods

### 2.1. Study Sites and Sampling Design

**Figure 1:**
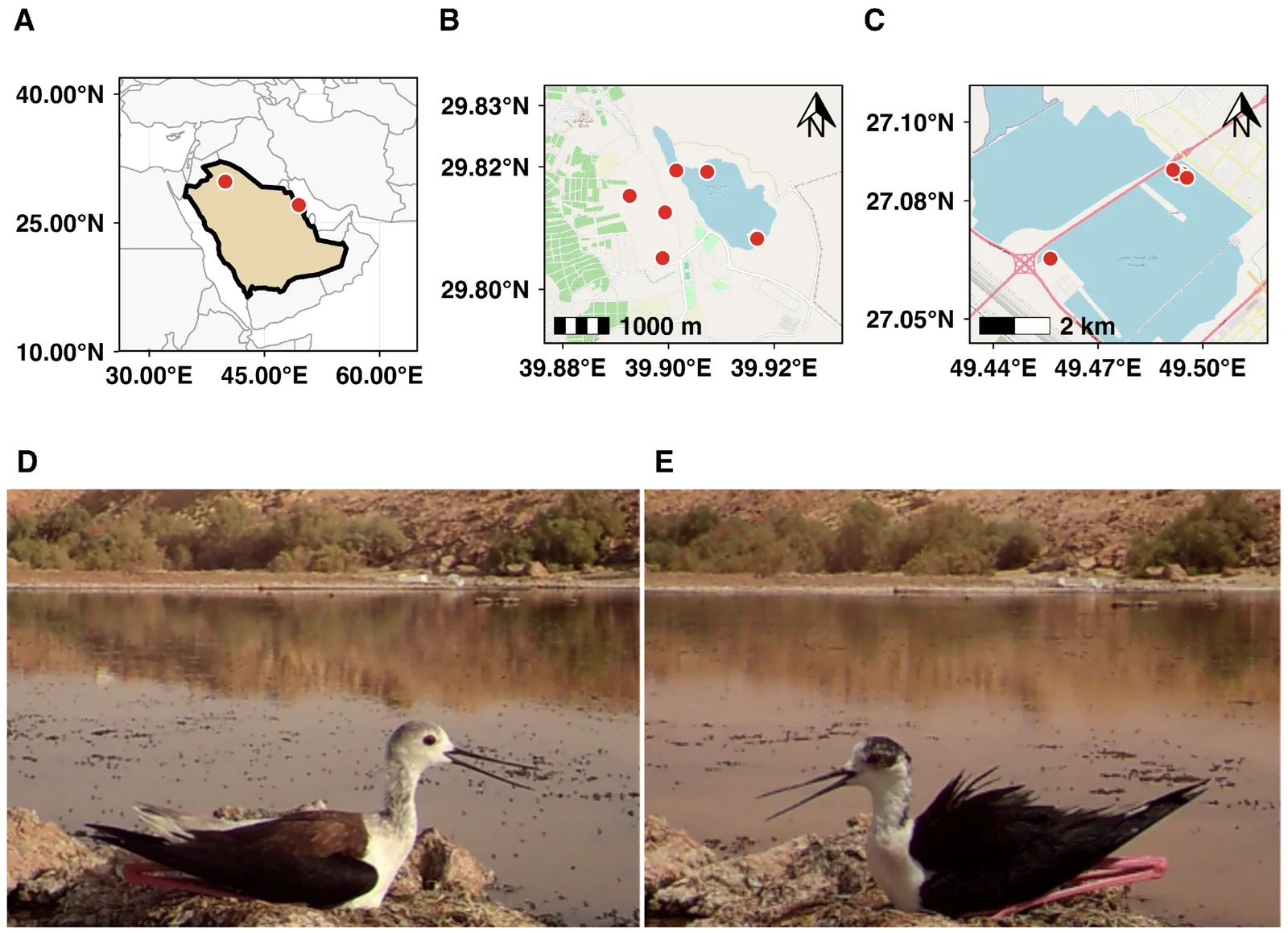
Study sites and sex-specific plumage of the Black-winged Stilt (*Himantopus himantopus*) in Saudi Arabia. (**A**) Location of Saudi Arabia within the Middle East, with red points indicating the two study sites. OpenStreetMap maps show the monitored nests (red circles) at (**B**) Dumat Al-Jandal in northwestern Saudi Arabia and (**C**) Sabkhat Al-Fasl on the eastern coast. Adult plumage differences are illustrated by (**D**) the female, characterized by brownish upperparts, and (**E**) the male, characterized by glossy black upperparts, particularly on the back and wings. Country boundaries in (**A**): Natural Earth (public domain). Map data in (**B**,**C**): © OpenStreetMap contributors (ODbL; https://www.openstreetmap.org/copyright) (accessed on 15 September 2026).

The study was conducted at two breeding sites of the Black-winged Stilt (*Himantopus himantopus*) in Saudi Arabia: Dumat Al-Jandal Lake in Al-Jouf Region, northern Saudi Arabia, and Sabkhat Al-Fasl southwest of Jubail Industrial City on the eastern coast (Figure 1). The two study sites were approximately 986 km apart. Dumat Al-Jandal Lake lies within the Dawmat al-Jandl wetland, northeast of Dumat Al-Jandal town. Until 1983–1984, the site comprised a medium-sized natural lake surrounded by extensive reed and sedge marshes (approximately 900 ha); much of the original wetland was subsequently drained and converted to agricultural land, with water redirected to an elevated reservoir of approximately 150 ha that now forms the principal water body [23]. The wetland was included in the avifaunal account of Al Jawf by Green [24] and is currently listed in the World Database of Key Biodiversity Areas [23]. The Sabkhat Al-Fasl recordings were obtained when the site comprised shallow artificial lagoons within an extensive sabkhah; the site characteristics at the time of sampling are described by AlRashidi and Shobrak [22]. Both sites supported breeding Black-winged Stilts. A total of 127 nests were recorded at Sabkhat Al-Fasl in 2016 [22], whereas field surveys at Dumat Al-Jandal recorded 22 nests in 2022 and 21 nests in 2023. Both sites are accessible to the public. All monitored nests were located in open areas near water. Nest-to-water distances ranged from 0.5 to 7 m at Dumat Al-Jandal (mean 2.67 m) and from 2 to 5 m at Sabkhat Al-Fasl (mean 3.83 m).

Twelve nests were included, six from each study group. At Dumat Al-Jandal, nests were located by observing adults returning to their nest sites, and nests were selected when the male and female could be reliably distinguished by plumage. Five Dumat Al-Jandal nests were monitored in 2022 and one in 2023. At Sabkhat Al-Fasl, the earlier field study located nests by walking along dykes and across the sabkhah; 16 nests were randomly selected for camera monitoring [22]. The six Sabkhat Al-Fasl nests included here were selected from those monitored in 2016 because males and females could be clearly distinguished from the images based on sex-specific plumage characteristics. Selection for this sex-specific analysis was therefore purposive rather than random. Nest coordinates (Table 1) were recorded with a handheld Garmin 12 GPS unit (Garmin International, Inc., Olathe, KS, USA). Because all Sabkhat Al-Fasl nests were sampled in 2016 whereas Dumat Al-Jandal nests were sampled in 2022–2023, study group and year were confounded. Study group was therefore retained as an adjustment factor, and its coefficient was interpreted as a contrast between the sampled groups rather than as an independent causal effect of locality or year.

Study-site maps were prepared in R using the sf and ggplot2 packages [25,26]. Country boundaries were obtained from Natural Earth [27], and map backgrounds for the two study sites were obtained from OpenStreetMap [28]. Nest locations were plotted on these backgrounds using WGS 84 coordinates (EPSG:4326).

### 2.2. Behavioral Monitoring and Scoring

Incubation behavior was recorded using Bushnell 8 MP Trophy Cam Black LED trail cameras (Bushnell Outdoor Products, Overland Park, KS, USA). For each nest, a continuous 24 h sequence was selected for behavioral scoring, beginning at least 2 h after camera installation and comprising 1440 images captured at one-minute intervals. In total, 17,280 images were obtained from the 12 nests. Recording start and end times are provided in Table 1. The number of calendar days between nest discovery and the start of the selected 24 h recording averaged 2.67 (range 0–7) at Dumat Al-Jandal and 0.83 (range 0–1) at Sabkhat Al-Fasl.

Cameras were installed at least 1.5 m from the nests in positions selected to provide a clear view of the nest while minimizing sunlight glare and disturbance to incubating birds. The position of each camera relative to the monitored nest is reported in Table 1. Exact camera azimuths were not measured in the field. Infrared imaging allowed nighttime monitoring without visible flashes that could disturb incubating birds. Camera installation was carried out with minimal disturbance, and adults generally returned to their nests within a few minutes. Ground-level temperature (°C) was recorded at one-minute intervals using a HOBO U12-012 data logger (Onset Computer Corporation, Bourne, Massachusetts, USA) placed adjacent to the nest, with recordings synchronized to camera timestamps. The monitoring protocol followed previous Black-winged Stilt work at Sabkhat Al-Fasl [22].

Sex was determined primarily from differences in adult upperpart plumage, with males exhibiting glossier black upperparts and females showing browner, less glossy upperparts. Repeated images of each pair facilitated individual recognition, with head and neck coloration used as secondary cues when necessary. Incubation attendance was scored for each image as 0 = no incubation, 1 = female incubation, or 2 = male incubation. Adults visible in the images but not incubating were recorded as non-incubating.

Body orientation was assessed along the longitudinal axis of the entire body rather than from head direction. Four cardinal directions (N, E, S, W) were used in the primary analyses, whereas eight compass directions (N, NE, E, SE, S, SW, W, NW) were used to examine the sensitivity of the results to finer directional resolution. The directional scoring procedure is illustrated schematically in Figure S1.

The two authors initially scored incubation attendance and body orientation in separate subsets of images and subsequently reviewed their scores together. To evaluate scoring consistency, one author subsequently reassessed the images without access to the previous records. Agreement was 100% for incubation attendance and 90.07% for four-direction body orientation (Cohen’s κ = 0.865). An error in the original directional reference for nest BWS_259 was identified and corrected.

### 2.3. Environmental Covariates and Directional Indices

Meteorological variables were derived from ERA5-Land Hourly and ERA5 Hourly reanalysis products and matched to the exact behavioral monitoring windows of each nest. Values were extracted at the coordinates of each nest through Google Earth Engine [29], using a scale parameter of 11,132 m for ERA5-Land and 27,830 m for ERA5. ERA5-Land provided 2 m air temperature, 2 m dew-point temperature, and the 10 m eastward and northward wind components (u10 and v10) [30], whereas total cloud cover was obtained from ERA5 Hourly [31]. Air and dew-point temperatures were converted from kelvin to °C, and ERA5-Land relative humidity was derived for each hour using the Magnus-type approximation of Alduchov and Eskridge [32]: RH = 100 × exp[(17.625 × Td)/(243.04 + Td) − (17.625 × T)/(243.04 + T)], constrained to 0–100%, where T and Td are air and dew-point temperatures in °C. Hourly timestamps were converted to Saudi Arabia local time (UTC+3) and assigned to the corresponding fixed two-hour clock periods; each two-hour nest-period therefore contained two ERA5 hourly observations. Hourly scalar wind speed was calculated as WS = sqrt(u10^2^ + v10^2^), and mean wind speed for each two-hour period was the arithmetic mean of the two hourly scalar speeds. Wind direction was derived separately from the two-hour vector mean rather than by averaging angular directions: WDfrom = [atan2(−mean(u10), −mean(v10)) × 180/π + 360] mod 360, giving the meteorological bearing from which the wind originated. Cloud cover and relative humidity were averaged over the same two-hour observations. ERA5-Land relative humidity was available for all 12 nests and was used in the complete-support models.

Solar altitude and azimuth were calculated for each one-minute record from its local date, time, and nest coordinates using suncalc [33]. Solar azimuths from suncalc were converted to north-referenced compass bearings (0° = north, increasing clockwise) by adding 180° after conversion to degrees and taking the result modulo 360°. Daylight was solar altitude >0°, and night was solar altitude ≤0°. Sun-related analyses used daylight observations only. The solar-avoidance index was −cos(θbird − θsun), ranging from −1 (toward the sun) to +1 (away from the sun), and wind alignment was cos(θbird − θwind-from), ranging from −1 (downwind) to +1 (toward the wind source). For the beta-binomial models, ‘away from sun’ denoted a solar-avoidance index >0, whereas ‘facing into wind’ denoted an absolute angular separation <90° from the wind-source direction.

### 2.4. Response Variables and Diel-Residualized Environmental Predictors

To quantify how incubation was partitioned between the sexes, male contribution was expressed as M/(M + F), where M and F denote the numbers of minutes incubated by males and females, respectively. Minutes during which neither parent incubated were excluded because they did not contribute to the division of incubation effort between the sexes. Female contribution was the exact complement of male contribution and was therefore not modeled separately.

Ground-level temperature exhibited strong diel variation. To separate residual environmental variation from the clock-time pattern already represented by the two-hour period factor, mean ground-level temperature was modeled as a function of nest identity and two-hour period across the sampled observations; the standardized residuals were then used as the ground-level temperature anomaly. Here, “anomaly” refers to a residualized departure within the sampled data set rather than deviation from a long-term nest-specific thermal baseline.

### 2.5. Statistical Analyses

#### 2.5.1. General Framework

All analyses were conducted in R 4.4.1 [34]. The package versions used in the final analysis were glmmTMB 1.1.14, DHARMa 0.4.7, emmeans 2.0.2, and survival 3.6-4. Proportional responses represented by paired counts were analyzed using beta-binomial generalized linear mixed models (GLMMs) with a logit link in glmmTMB [35]. Nest identity was included as a random intercept [36], and orientation models also accounted for repeated sex-specific summaries within nest-periods. Continuous environmental predictors were standardized before model fitting so that coefficients were comparable on a one-standard-deviation scale. Because each nest was observed during only one 24 h period, the independent biological replication comprised 12 nests, whereas the recordings spanned eight distinct meteorological monitoring windows, with some nests monitored concurrently.

#### 2.5.2. Overall Sex Partition and Daylight-Night Contrast

To test whether the nest-level male share differed from 0.5 over 24 h, male share was calculated independently for each nest and evaluated with an exact two-sided nest-level sign-flip randomization test. The procedure enumerated all possible sign assignments around the null and therefore based inference on the 12 independent nests rather than the 17,280 one-minute records [37]. A whole-nest nonparametric bootstrap provided an approximate percentile 95% confidence interval for the nest-mean male share [38]. This was a test of departure from 0.5, not an equivalence test.

To test whether the relative contributions of the sexes differed between daylight and night, male share was first summarized separately for daylight and night within each nest. The within-nest daylight-minus-night difference was tested with an exact paired nest-level sign-flip randomization test and summarized with an approximate whole-nest bootstrap confidence interval [37,38]. The paired exact test was used as the primary inferential test because it directly reflects the 12 independent within-nest contrasts.

#### 2.5.3. Two-Hour Diel Pattern and Environmental Associations with Sex Partition

To test whether the relative contributions of males and females varied among the 12 two-hour periods, a parsimonious beta-binomial GLMM was fitted to male and female incubation counts with period and study group as fixed effects and nest as a random intercept [39]. The global contribution of period was assessed by comparing the full model with the otherwise identical model lacking the period factor using a likelihood-ratio test (LRT; df = 11). Because asymptotic likelihood-ratio *p*-values may be unreliable with only 12 nests and residual underdispersion, the test was calibrated by a 999-replicate parametric bootstrap under the reduced model.

An additional within-nest cyclic-shift Monte Carlo sensitivity analysis with 4999 shifts was used to evaluate the robustness of the clock-time effect. This analysis circularly shifted each nest’s complete 12-period male-share profile independently relative to absolute clock time. It therefore preserved the within-nest temporal sequence and total sex allocation while breaking alignment with absolute clock time. The cyclic-shift analysis was interpreted as a distinct sensitivity test of the same clock-time pattern, not as independent replication [37].

To examine environmental associations with sex partition after accounting for diel period, a single mutually adjusted beta-binomial GLMM included period, study group, ground-level temperature anomaly, wind speed, ERA5-Land relative humidity, and cloud cover, with nest as a random intercept. Each environmental predictor was evaluated by a drop-one likelihood-ratio test. The four environmental *p* values were adjusted using the Holm procedure [40]. Whole-nest bootstrap confidence intervals and family-wise adjusted intervals were examined as supplementary measures of coefficient stability. These environmental associations were regarded as exploratory rather than causal, particularly because nests monitored within the same weather window shared reanalysis conditions. Model-based marginal estimates for figures were obtained using the emmeans package [41].

#### 2.5.4. Solar-Relative Orientation

Daylight solar-relative orientation was summarized by nest, sex, and two-hour period as the number of incubated minutes directed away from versus toward the sun. A beta-binomial GLMM tested the sex term after adjusting for two-hour period and study group, with random intercepts for nest and nest-period. The prespecified primary analysis used verified four-cardinal body directions. A matched eight-cardinal model, nest-bootstrap contrasts, and comparisons adjusted for each nest-by-sex baseline compass distribution examined sensitivity to directional resolution and fixed nest preferences.

#### 2.5.5. Wind-Relative Orientation

Wind-relative orientation was summarized by nest, sex, and two-hour period as paired counts facing into the wind-source half-plane versus other orientations. A beta-binomial GLMM included two-hour period, study group, sex, standardized wind speed, and the sex × wind-speed interaction, with random intercepts for nest and nest-period. A joint two-degree-of-freedom likelihood-ratio test evaluated the sex main effect and the sex × wind-speed interaction together; a separate one-degree-of-freedom likelihood-ratio test evaluated the interaction alone. Both are nominal, model-based tests. We repeated these tests using the verified eight-cardinal classifications and, as a focused leave-one-nest-out sensitivity check, after excluding BWS_259, for which the historical image-to-compass reference had been corrected. Additional analyses accounted for shared meteorological monitoring windows and nest-by-sex compass preferences because minute-level images and shared weather records were not independent replicates.

As exploratory robustness checks, the wind models were refitted after excluding periods with mean wind speed below 2 m s^−1^, directional steadiness below 0.90, or both. Directional steadiness was calculated as the magnitude of the mean wind vector divided by the mean scalar wind speed. These cutoffs were sensitivity thresholds rather than established biological response thresholds.

#### 2.5.6. Compass-Preference and Orientation-Episode Sensitivity Analyses

To assess whether apparent sex differences in solar- or wind-relative orientation could arise from fixed compass preferences, each nest-by-sex directional distribution was compared with its observed north, east, south, and west frequencies. Whole-nest and shared-monitoring-window summaries, exact paired sign-flip tests, cardinal-rotation nulls, and orientation-episode analyses provided complementary sensitivity checks. An orientation episode was defined as a run of consecutive incubation minutes with the same sex and verified four-cardinal body direction. These analyses assessed the robustness of sex comparisons rather than establishing causal responses to the sun or wind.

Four exploratory episode-onset conditional-choice models (solar-only, wind-only, joint-angle, and joint-horizontal) evaluated the selected cardinal direction while adjusting for each nest-by-sex combination’s baseline compass distribution. These conditional logistic models were fitted using survival::clogit [42,43]. The joint-horizontal model projected solar alignment onto the horizontal plane. Sex-by-alignment effects were summarized using 95% percentile cluster-bootstrap intervals, with 999 resamples for each single-cue model and 499 for each joint model. Resampling was conducted at the nest level for the solar-only model (n = 12) and at the shared meteorological monitoring-window level for wind-related models (n = 8). These models were treated as exploratory sensitivity analyses rather than independent confirmatory evidence.

#### 2.5.7. Multiplicity, Robustness, and Model Diagnostics

The two primary wind tests (joint sex-related terms and the sex × wind-speed interaction) were reported separately as nominal model-based tests. The additional analyses described above were treated as sensitivity checks rather than independent confirmatory findings. Model-based confidence intervals were distinguished from bootstrap intervals, and nonsignificance was not interpreted as evidence of sex equivalence.

Model adequacy was evaluated with simulation-based DHARMa residual diagnostics using 1000 simulations, including both unconditional and conditional simulations of the random effects [44]. Checks of convergence and descriptive within-nest residual lag correlations were conducted. Exploratory diagnostic checks compared models with and without an AR(1) temporal term and assessed sensitivity to local apparent solar time, shared weather-monitoring windows, and within-nest centering of environmental variables; none replaced the primary models. Exact sign-flip tests were two-sided, and *p* < 0.05 was used as the conventional significance threshold.

## 3. Results

### 3.1. Sampling Completeness and Incubation Attendance

All 12 nests contributed a complete 24 h sequence of 1440 one-minute records (17,280 records total; Table 1), spanning the two study groups shown in Figure 1. Across all minutes, females incubated for 7861 min (45.49%), males for 8059 min (46.64%), and no incubation was recorded for 1360 min (7.87%). All 15,920 incubation minutes had a scorable cardinal body direction. Nest-level incubation totals and the contributions of each sex are presented in Table S1.

### 3.2. Sex-Specific Partitioning Across the Diel Cycle

When summarized at the nest level, males contributed a mean 50.72% of incubation minutes (approximate 95% nest-bootstrap CI 47.21–54.35%). The exact sign-flip test provided no clear evidence that the nest-mean male share differed from 0.5 (*p* = 0.697; Table 2). The mean daylight-minus-night difference in male share was −9.22 percentage points (approximate 95% bootstrap CI −22.65 to +5.51 percentage points), and the paired contrast was inconclusive (exact *p* = 0.234; Table 2).

**Figure 2:**
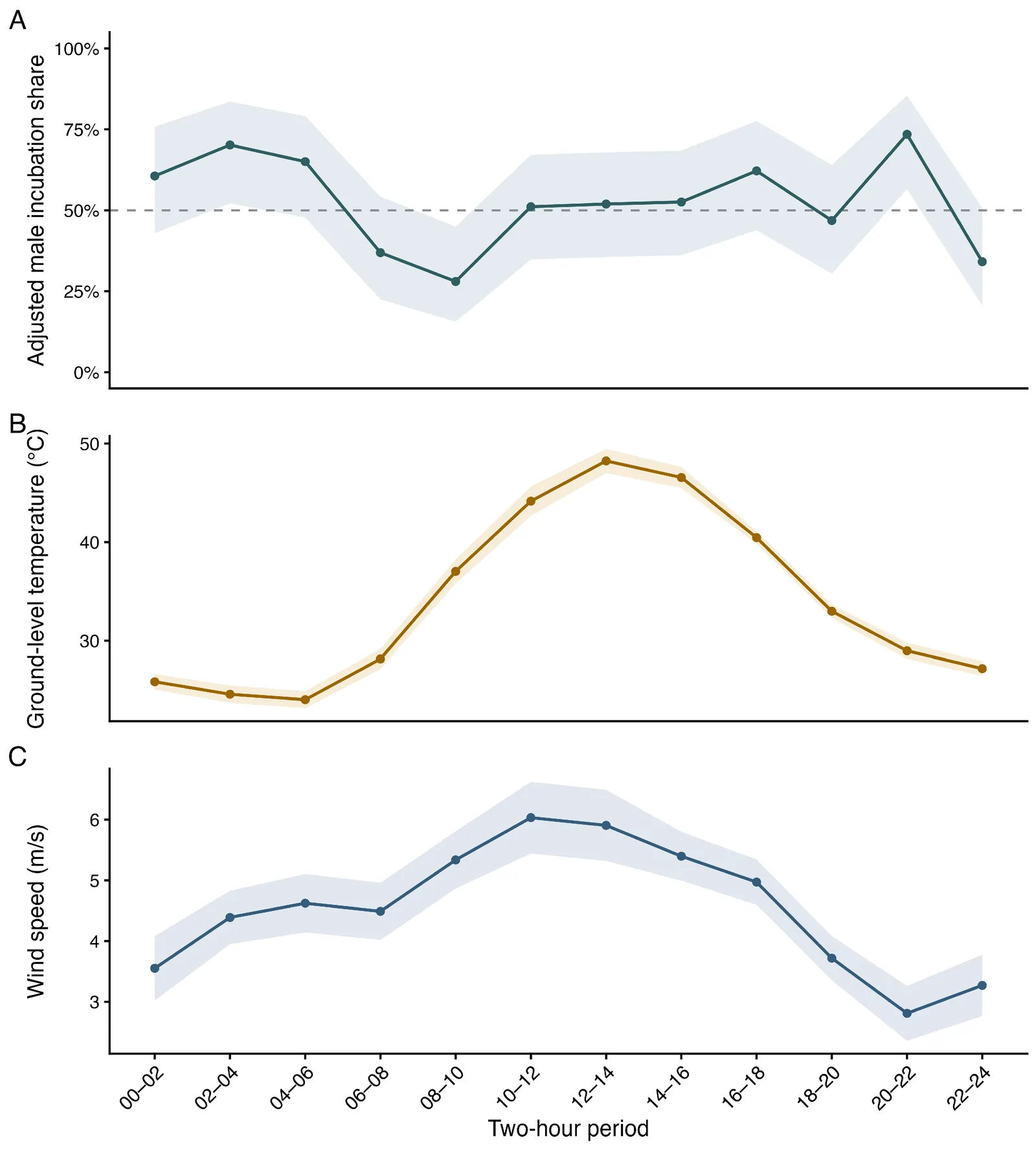
Diel patterns in male incubation share and environmental conditions across 12 two-hour periods. (**A**) Model-adjusted male share with 95% model confidence intervals and a 50% reference; (**B**) mean ground-level temperature; (**C**) mean wind speed. Environmental panels are descriptive.

Male contribution varied among the two-hour periods (global beta-binomial GLMM: χ^2^ = 28.33, df = 11; nominal *p* = 0.0029; parametric-bootstrap *p* = 0.004; Figure 2A; Table 2). A within-nest cyclic-shift test also supported alignment with clock time (4999 shifts, *p* = 0.042; Table 2). Descriptively, average male share was lowest at 22:00–24:00 (31.9%) and highest at 20:00–22:00 (71.2%) across the 12 nest-level period summaries; period-specific values were heterogeneous between nests (Table S2; Figure S2). Mean ground-level temperature and wind speed followed diel patterns (Figure 2B,C), but these descriptive trends should not be equated with environmental effects on the sex division of care.

### 3.3. Environmental Associations with Sex Partition

After accounting for the two-hour period and study group in the mutually adjusted model based on all 12 nests, none of the four environmental predictors showed a clear association with male share after Holm correction (Table 3; Figure S3). The estimated coefficients were −0.046 for ground-level temperature anomaly (Holm-adjusted *p* = 1.000), 0.071 for wind speed (Holm-adjusted *p* = 1.000), 0.352 for ERA5-Land relative humidity (Holm-adjusted *p* = 0.308), and −0.113 for cloud cover (Holm-adjusted *p* = 1.000).

### 3.4. Sex Differences in Orientation Relative to Solar Position

**Figure 3:**
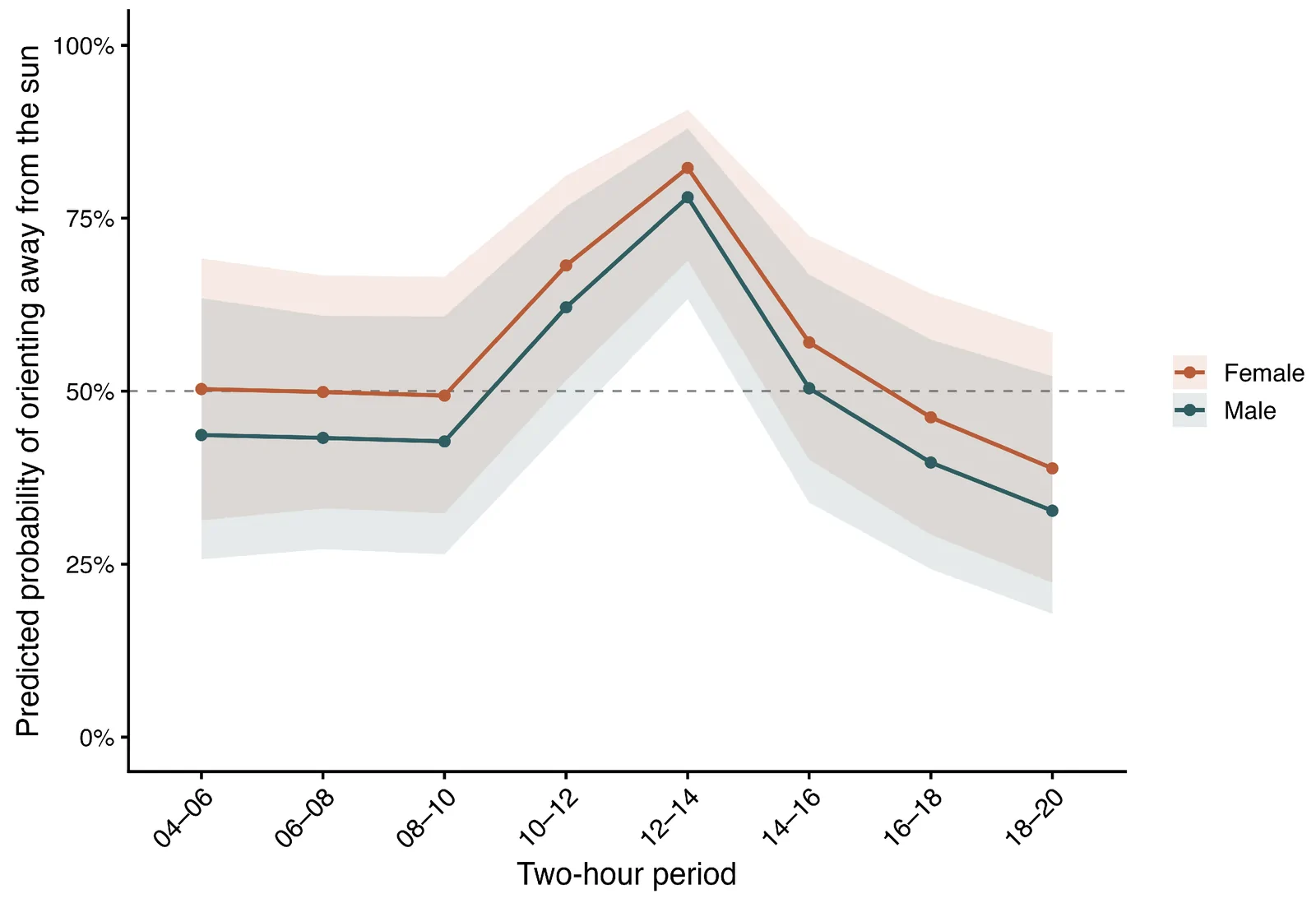
Model-adjusted probability of body orientation away from the sun by sex across daylight two-hour periods, using verified four-cardinal classifications; nominal 95% model confidence intervals. The 50% line is a descriptive reference and does not establish active solar avoidance.

With verified four-cardinal directions, the daylight solar-relative model provided no clear evidence of a male–female difference after adjustment for two-hour period and study group (β = −0.267, OR = 0.766, 95% model-based CI for the OR 0.531–1.103; LRT χ^2^ = 2.036, df = 1, *p* = 0.154; Table 4; Figure 3). The matched exploratory eight-direction model was also inconclusive (*p* = 0.675). Predicted probabilities by sex and daylight period are provided in Table S3.

After accounting for the baseline compass distribution of each sex within each nest, the male-minus-female difference in excess solar alignment was −0.080 (approximate bootstrap 95% CI −0.219 to 0.069; exact nest sign-flip *p* = 0.316). The episode-weighted analysis (*p* = 0.167) and cardinal-rotation test (*p* = 0.164) were also inconclusive (Table 4).

### 3.5. Sex Differences in Orientation Relative to Wind Direction

**Figure 4:**
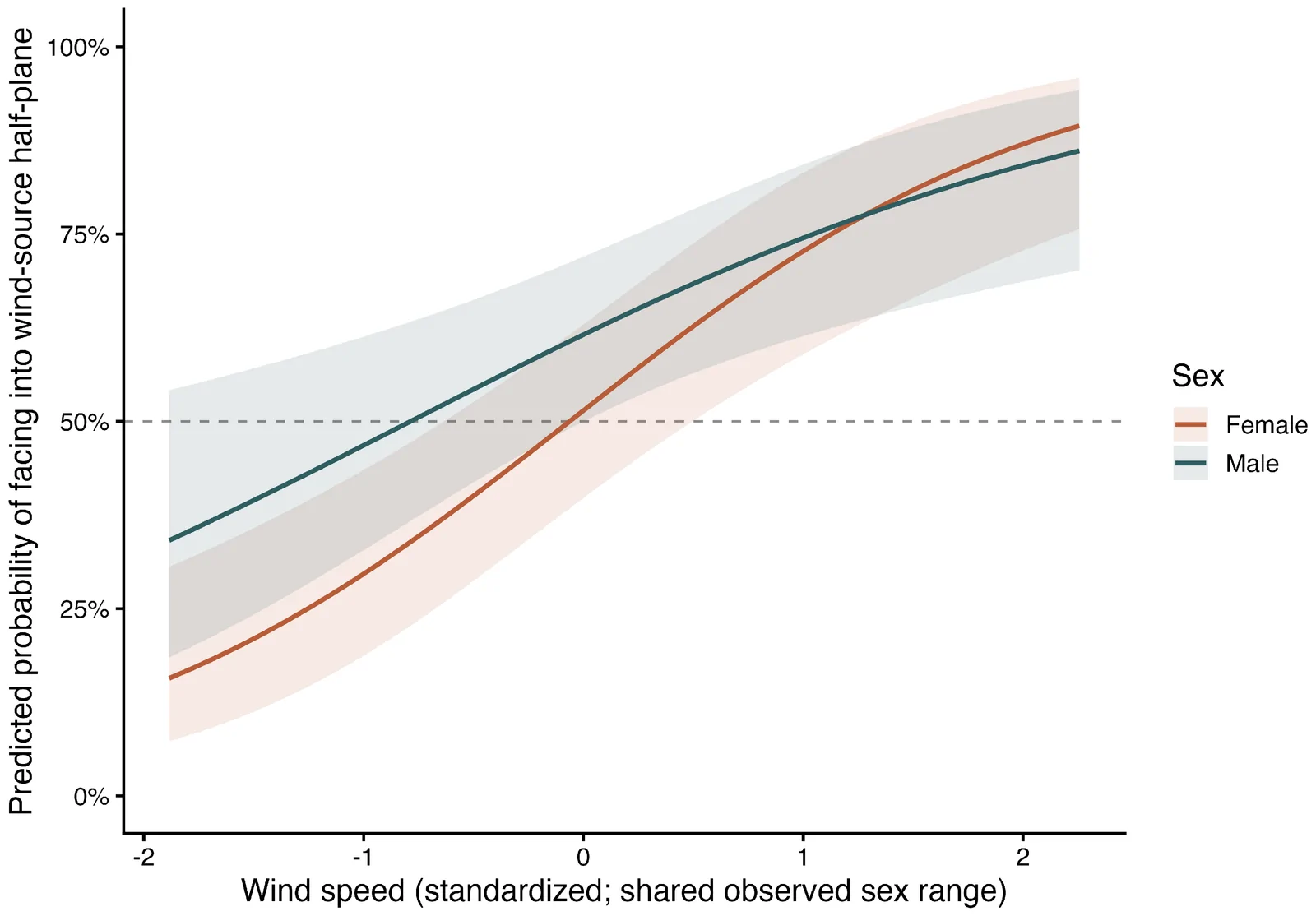
Model-predicted probabilities of facing into the wind by sex and standardized wind speed using the verified four-cardinal classification, with nominal model confidence intervals. A difference in fitted probabilities at selected speeds does not establish a sex × wind-speed interaction. The nominal sex-related findings were not consistently corroborated in compass-adjusted and episode-based analyses.

The primary verified four-direction wind model suggested an association between sex and wind-facing in the nominal joint test (χ^2^ = 8.372, df = 2, *p* = 0.015; Figure 4). This nominal finding was not consistently corroborated by compass-preference-adjusted and episode-based sensitivity analyses and does not establish a sex-specific wind response (Table 5). At mean standardized wind speed, the model’s conditional male–female contrast was β = 0.414 (OR = 1.513, 95% model-based CI for the OR 1.066–2.146; Wald-test *p* = 0.020). However, the sex × wind-speed interaction was inconclusive (β = −0.323, OR = 0.724, 95% model-based CI for the OR 0.510–1.028; LRT χ^2^ = 3.256, df = 1, *p* = 0.071). The matched exploratory eight-direction model also yielded a nominal joint sex-related difference (*p* = 0.038), whereas its sex × wind-speed interaction was inconclusive (*p* = 0.172). Representative model predictions are provided in Table S4.

After adjustment for nest-by-sex compass preferences, the male–female contrast in wind alignment was inconclusive (exact *p* = 0.414), as was the sex difference in its wind-speed slope (exact *p* = 0.266; Table 5). An exploratory compass-rotation test of the sex difference in the wind-speed alignment slope was also inconclusive (*p* = 0.139).

Exploratory refits excluding weak or directionally unsteady wind periods retained nominally significant joint sex-related tests (Table S5). The nominal joint finding also persisted after excluding the directionally corrected nest BWS_259, whereas the sex × wind-speed interaction remained inconclusive.

### 3.6. Orientation Sensitivity Checks

A total of 1374 four-cardinal orientation episodes were identified (Table S6). Exploratory episode-onset conditional-choice analyses yielded mixed results: several sex-related 95% cluster-bootstrap confidence intervals excluded zero, whereas separate exact compass-adjusted tests were inconclusive for solar alignment (*p* = 0.218), wind alignment (*p* = 1.000), and the sex difference in the wind-speed slope (*p* = 0.094; Table S7).

### 3.7. Diagnostic Findings

An exploratory cyclic-shift analysis using local apparent solar time yielded *p* = 0.0626, compared with *p* = 0.042 using local clock time. DHARMa diagnostics indicated underdispersion in the two-hour-period and environmental male-share models under both unconditional and conditional simulations (dispersion ratios approximately 0.68; all *p* < 0.001; Table S5).

An exploratory AR(1) model estimated weak within-nest temporal correlation (ρ = 0.0255) and had a lower AIC than the comparable model without AR(1) (1336.83 versus 1342.59), but underdispersion persisted. Conditional DHARMa diagnostics did not indicate clear dispersion departures for the solar- or wind-orientation models (*p* = 0.682 and *p* = 0.212, respectively), although the unconditional wind-model diagnostic suggested underdispersion (*p* = 0.036; Table S5).

## 4. Discussion

### 4.1. Diel Partitioning of Incubation Between the Sexes

Both sexes contributed substantially to incubation over 24 h, but male share varied across two-hour periods. The broader daylight–night comparison did not provide clear evidence of a difference, and the near-equal daily contributions should not be interpreted as evidence of statistical equivalence between the sexes.

Cuervo [21] provided an important foundation for understanding biparental care in Black-winged Stilts by demonstrating that male and female parental roles change across reproductive stages. Males invested more time in nest building before and during egg laying, whereas females increased their nest attendance as incubation progressed. These findings highlighted the complementary contributions of both parents and the importance of reproductive stage in shaping parental investment. Our study builds on this work by examining the temporal division of incubation within a complete 24 h cycle. Cuervo [21] also examined time-of-day variation during daylight observations but found no statistically significant effect on incubation attendance across five daytime periods. In contrast, our study detected variation in male incubation share across 12 two-hour periods spanning 24 h. This difference should not be interpreted as a contradiction, given differences in sampling design, temporal resolution, study populations, and reproductive-stage coverage. Instead, the two studies provide complementary evidence that parental roles may vary across reproductive stages and within individual days.

Similar distinctions between total daily incubation effort and its temporal distribution have been reported in shorebirds [14,15]. Kentish Plovers (*Charadrius alexandrinus*) breeding in a hot Arabian environment likewise showed sex-specific changes in incubation schedules across two-hour intervals [9]. Huang et al. [12] further showed that Kentish Plover males changed the timing of their attendance even when their total daily attendance remained unchanged. In Northern Lapwings (*Vanellus vanellus*), male incubation contributions varied substantially among nests and were concentrated around sunrise and sunset [45].

The cyclic-shift result was sensitive to the time reference used, and variation among nests and residual underdispersion caution against inferring a universal incubation schedule across pairs. Such temporal partitioning may reflect interdependent parental decision-making, because the effort of one parent can influence the contribution of its partner [6,46].

### 4.2. Environmental Conditions and Incubation Allocation

Environmental conditions may influence both the incubation effort required to maintain suitable thermal conditions for the eggs and how that effort is divided between the sexes. Studies on the Kentish Plover illustrate how such relationships vary with ecological context. In a hot environment, male incubation increased with ambient temperature during midday periods [9], while experimental removal of nest cover increased incubation effort and changeover frequency [10]. In exposed, but not covered, Kentish Plover nests, male daytime incubation also became more likely as ambient temperature increased [47]. Hunter et al. [11] reported a negative association between daytime nest attendance and temperature at an undisturbed site, whereas Boughdiri et al. [13] found no evidence that nest cover predicted either total incubation or male incubation share in a Mediterranean population. These contrasting findings suggest that environmental associations may depend on population, thermal setting, and the component of parental care measured.

Male and female parents at the same nest share the local thermal environment at any given time, but their cumulative exposure may differ because they incubate at different times. High thermal demands might increase the need for biparental cooperation without requiring a 50:50 division of duties. Previous observations of Black-winged Stilts in eastern Saudi Arabia documented high nest attendance during periods of intense heat [22], although overall nest attendance does not indicate how incubation is divided between the sexes. In our study, none of the measured environmental variables showed a clear association with male incubation share after accounting for diel period and multiple testing. These findings do not establish that temperature or the other measured environmental conditions explain the near-equal daily contributions.

### 4.3. Sex-Specific Solar-Relative Body Orientation

After complete directional rescoring, there was no clear evidence of a sex difference in solar-relative orientation in either the primary four-direction or exploratory eight-direction model. Compass-preference-adjusted, equal-episode-weight, and rotation-null analyses did not provide clear support for such a difference. Earlier observations in Black-winged Stilts described apparent orientation away from the sun [22], but our sex-focused analyses did not establish a difference in solar-relative body orientation between males and females. Moreover, as the sun’s position changes, a fixed compass preference can alter apparent solar-relative alignment.

Solar-related body orientation and posture may affect radiative heat gain [18,48,49,50,51], but our data did not include measurements of adult body temperature, egg thermal exposure, or radiative flux. Therefore, the observed body orientations cannot establish a thermoregulatory function.

### 4.4. Sex-Specific Wind-Relative Body Orientation

The primary four-direction analysis suggested a nominal sex-related difference in wind-relative body orientation, with a similar finding in the exploratory eight-direction analysis. However, these model-based findings were not consistently corroborated by compass-preference-adjusted and episode-based sensitivity analyses; thus, they do not establish a sex-specific response to wind. These findings persisted after excluding BWS_259, for which the original directional reference had been corrected. At mean wind speed, the primary model estimated higher odds of facing into the wind for males than females, but this conditional model contrast should not be interpreted as confirmatory evidence. Neither directional analysis provided clear evidence of a sex × wind-speed interaction. Thus, the results do not establish that male and female responses diverged with increasing wind speed.

Wind-related orientation during incubation has been documented in Common Terns (*Sterna hirundo*) [17], White Storks (*Ciconia ciconia*) [52], and Crowned Plovers (*Vanellus coronatus*) [53]. Facing wind may serve mechanical or thermoregulatory functions, but these studies concern responses to wind, whereas our primary question concerned differences between sexes. Similarly, resting shorebirds have been observed facing into the wind in relation to wind speed [54], and orientation and posture can affect thermal conditions in birds [18,55]. These findings provide biological context but do not establish that wind caused active realignment in our sampled nests.

Several nests shared reanalysis wind estimates, leaving only eight distinct monitoring windows. Moreover, fixed nest-specific compass preferences may produce apparent wind alignment without active reorientation. Thus, the nominal sex-related wind differences should be interpreted as model-dependent rather than definitive evidence of an active sex-specific response to wind.

Exploratory exclusions of weak or directionally unsteady wind periods retained the nominal joint sex-related findings but did not provide clear evidence of a sex × wind-speed interaction. Because the filtering thresholds were not biologically validated and directional steadiness was estimated from only two hourly reanalysis vectors per period, these sensitivity checks do not establish a biological wind-response threshold.

### 4.5. Interpreting Directional Orientation and Sensitivity Analyses

Although several exploratory conditional-choice bootstrap intervals excluded zero (Table S7), compass-preference-adjusted, rotation-null, and exact monitoring-window analyses did not consistently support the nominal sex-related wind findings. This discrepancy indicates that inference depends on modeling assumptions and how highly repeated observations are aggregated.

Exact compass-adjusted episode-onset tests provided no clear evidence of sex differences in solar-relative or wind-relative alignment, or in the wind-speed slope. However, successive orientation episodes may not be independent, and neither the exact tests nor the exploratory bootstrap results establish active sex-specific reorientation in response to environmental cues.

### 4.6. Limitations

Each nest contributed only one 24 h sequence, preventing assessment of repeatability across days and incubation stages. Clutch age and incubation stage were not recorded, limiting biological interpretation. Although 17,280 images were analyzed, the independent sampling units comprised 12 nests and, for shared wind conditions, eight monitoring windows. Study site and sampling year were confounded (Sabkhat Al-Fasl, 2016; Dumat Al-Jandal, 2022–2023), and nests were partly selected based on reliable sex identification. Because blind rescoring was performed by one of the original scorers, the reported agreement reflects rescoring consistency rather than independent interobserver agreement. Camera positions relative to nests were verified, but exact camera azimuths were not measured.

Environmental measurements differed in resolution and coverage. Ground-level temperature was recorded at nests, while wind, cloud cover, and reanalysis relative humidity came from hourly products. Thus, reanalysis wind direction may not reproduce nest-scale airflow. Four-cardinal body-axis coding could obscure angular detail; eight-cardinal rescoring was assessed, but some octants were rare, and the same limited number of nests constrained inference.

The two-hour and environmental male-share models retained underdispersion in both conditional and unconditional residual simulations. Although parametric-bootstrap and cyclic-shift analyses supported the diel period pattern, potential model misspecification cannot be excluded. Future studies should monitor more nests across repeated days, with verified camera bearings and direct nest-level wind and egg-temperature measurements.

## 5. Conclusions

Male and female Black-winged Stilts showed near-equal mean incubation contributions over 24 h, but male share varied across two-hour periods, revealing differences in the timing of parental contributions that were not apparent from daily totals. None of the measured environmental predictors showed a clear association with male incubation share after correction for multiple testing, and solar-relative orientation showed no clear sex difference. The wind-facing results were not consistently supported by sensitivity analyses and did not establish a sex-specific response to wind. The sex × wind-speed interaction was not clearly supported, and the results do not establish active sex-specific reorientation in response to increasing wind speed. Further studies involving more independent nests and monitoring periods, repeated observations across days, and direct nest-level airflow measurements are needed to clarify these relationships.

## Figures and Tables

**Table 1:** Sampling design, nest coordinates, camera positions, and 24 h acquisition periods for the 12 Black-winged Stilt nests.

Nest ID	Study Group	Year	Latitude	Longitude	Acquisition Start	Acquisition End	Duration (h)	1 min Records	Camera Position
BWS01	Dumat Al-Jandal	2022	29.808250	39.916778	2022-05-25 12:00	2022-05-26 11:59	24	1440	W
BWS02_1	Dumat Al-Jandal	2022	29.819389	39.901472	2022-05-30 12:00	2022-05-31 11:59	24	1440	N
BWS015	Dumat Al-Jandal	2022	29.805083	39.898889	2022-05-28 20:00	2022-05-29 19:59	24	1440	N
BWS016	Dumat Al-Jandal	2022	29.815250	39.892667	2022-05-28 20:21	2022-05-29 20:20	24	1440	W
BWS018	Dumat Al-Jandal	2022	29.812556	39.899361	2022-06-12 19:24	2022-06-13 19:23	24	1440	W
BWS406	Dumat Al-Jandal	2023	29.819175	39.907330	2023-06-13 12:00	2023-06-14 11:59	24	1440	S
BWS_259	Sabkhat Al-Fasl	2016	27.065333	49.456364	2016-06-02 14:00	2016-06-03 13:59	24	1440	N
BWS304_1	Sabkhat Al-Fasl	2016	27.087006	49.493989	2016-06-04 00:00	2016-06-04 23:59	24	1440	N
BWS406_1	Sabkhat Al-Fasl	2016	27.086931	49.494092	2016-06-04 00:00	2016-06-04 23:59	24	1440	S
BWS_614	Sabkhat Al-Fasl	2016	27.086767	49.492792	2016-07-13 16:00	2016-07-14 15:59	24	1440	E
BWS_618	Sabkhat Al-Fasl	2016	27.087831	49.491489	2016-07-13 16:00	2016-07-14 15:59	24	1440	S
BWS_627	Sabkhat Al-Fasl	2016	27.085844	49.495472	2016-07-13 16:00	2016-07-14 15:59	24	1440	S

Note. All timestamps are Saudi Arabia local time (UTC+3). Each nest contributed a complete 24 h record of 1440 one-minute observations.

**Table 2:** Sex-specific incubation attendance and diel contrasts.

Result	Independent Nests	Estimate	95% CI	*p*	Inference
Nest-mean male share among incubation minutes	12	50.72%	47.21 to 54.35%	0.697	Exact sign-flip; approximate nest bootstrap CI
Male-share difference: daylight minus night	12	−9.22 pp	−22.65 to +5.51 pp	0.234	Exact paired sign-flip; approximate nest bootstrap CI
Global two-hour-period effect	12	χ^2^ = 28.33; df = 11	—	0.004	Beta-binomial GLMM; 999/999 parametric-bootstrap LRT; nominal *p* = 0.0029
Global two-hour-period effect: sensitivity	12	4999 cyclic shifts	—	0.042	Within-nest clock-time cyclic-shift test

Note. pp = percentage points. Overall and daylight–night *p* values are exact nest-level sign-flip tests; intervals are approximate percentile whole-nest bootstrap CIs. The two-hour likelihood-ratio test (χ^2^ = 28.33, df = 11) was calibrated by 999 successful parametric-bootstrap fits. The 4999-shift test is a sensitivity analysis of the same clock-time hypothesis.

**Table 3:** Environmental associations with the male share of incubation attendance.

Support	Predictor	β	SE	LRT *p*	Holm *p*	Nominal Model OR (95% CI)	LRT χ^2^ (df = 1)
All nests	Ground-level temperature anomaly	−0.046	0.111	0.678	1.000	0.955 (0.768–1.187)	0.172
All nests	Wind speed	0.071	0.139	0.610	1.000	1.074 (0.817–1.411)	0.260
All nests	Relative humidity (ERA5-Land)	0.352	0.199	0.077	0.308	1.422 (0.963–2.099)	3.127
All nests	Cloud cover	−0.113	0.132	0.393	1.000	0.893 (0.690–1.157)	0.729

Note. OR = odds ratio; SE = standard error; LRT = likelihood-ratio test; Holm *p* = Holm-adjusted *p* value. Coefficients are logit-scale estimates from a mutually adjusted beta-binomial GLMM; continuous environmental predictors were standardized. All four factors were tested by single-degree-of-freedom drop-one LRTs with Holm correction. Model-based odds-ratio intervals are nominal and must not be treated as family-wise significant; multiplicity-adjusted bootstrap intervals did not exclude zero for any predictor.

**Table 4:** Sex differences in solar-relative body orientation and sensitivity analyses.

Analysis	Sex Contrast	Estimate	95% CI	*p*	N	Check
Primary GLMM (verified 4 directions)	Male versus female	−0.267 (0.766)	OR 0.531–1.103	0.154	12	Adjusted; nominal LRT
Compass-adjusted	Male minus female	−0.080	−0.219 to +0.069	0.316	12	Exact nest sign-flip
Equal episode weight	Male minus female	−0.055	−0.122 to +0.022	0.167	12	Sensitivity
Rotation-null	Sex difference	—	—	0.164	12	999 rotations

Note. The primary solar comparison is the male–female contrast based on the verified four-cardinal classifications after adjustment for two-hour period and study group. The eight-cardinal analysis and compass-preference and episode-based tests are exploratory sensitivities, not tests of overall sun avoidance. CIs are model-based or approximate bootstrap intervals as indicated. In the primary GLMM row, the estimate is β (OR); sensitivity estimates are contrasts on their respective scales.

**Table 5:** Sex differences in wind-relative body orientation and sensitivity analyses.

Analysis/Predictor	Estimate	95% CI	*p*	N (Nests/Windows)	Inference
Joint sex-related wind test	χ^2^ = 8.372; df = 2	—	0.015	12/8	Primary nominal LRT, 4 directions
Sex × wind-speed interaction	β = −0.323; OR = 0.724	OR 0.510–1.028	0.071	12/8	One-df LRT (χ^2^ = 3.256)
Sex contrast at mean wind speed	β = 0.414; OR = 1.513	OR 1.066–2.146	0.020	12/8	Conditional Wald test
Compass-adjusted wind contrast	+0.046	−0.057 to +0.130	0.414	8 windows	Exact window sign-flip
Equal orientation-episode weight	−0.048	−0.125 to +0.030	0.297	8 windows	Exploratory episode sensitivity
Compass-adjusted sex × wind slope	−0.085	−0.211 to +0.034	0.266	8 windows	Exact window sign-flip
Rotation-null sex difference	—	—	0.410	8 windows	999 cardinal rotations

Note. Primary tests use verified four-cardinal data. The joint two-df LRT tests the sex term and its interaction with standardized wind speed together; it is distinct from the one-df interaction test. The eight-direction and omitted-nest models provide exploratory robustness checks. Nominal model *p* values are not bootstrap- or cluster-adjusted and must not be interpreted as though minute-level observations were independent replicates. Weather observations were shared across eight monitoring windows. The nominal sex-related wind findings were not consistently corroborated by compass-preference-adjusted and episode-based sensitivity analyses.

## Data Availability

The data presented in this study are available from the corresponding author upon reasonable request.

## Supplementary Materials

The following supporting information can be downloaded at: https://www.mdpi.com/article/10.3390/ani16213467/s1, Table S1. Nest-level incubation attendance and sex-specific contributions; Table S2. Mean male incubation share across two-hour periods; Table S3. Adjusted solar-relative orientation probabilities by sex and daylight period; Table S4. Representative sex-specific wind-facing probabilities at selected wind speeds; Table S5. Simulation-based model diagnostics and wind-filter sensitivity; Table S6. Compass-direction distributions and directional changes by nest and sex; Table S7. Episode-onset and conditional-choice sensitivity analyses of sex differences in orientation; Figure S1. Schematic of camera position and four- and eight-direction body-axis classification; Figure S2. Nest-specific two-hour profiles of male incubation share with color-coded incubation attendance; Figure S3. Adjusted environmental associations with male incubation share.

## References

[1] Clutton-Brock T.H. (1991). The Evolution of Parental Care.

[2] Reynolds J.D., Goodwin N.B., Freckleton R.P. (2002). Evolutionary transitions in parental care and live bearing in vertebrates. Philos. Trans. R. Soc. Lond. B Biol. Sci..

[3] Wang D., Zhang W., Yang S., Li Richter X.-Y. (2023). Sex differences in avian parental care patterns vary across the breeding cycle. Nat. Commun..

[4] Webb D.R. (1987). Thermal tolerance of avian embryos: A review. Condor.

[5] Reid J.M., Monaghan P., Nager R.G., Deeming D.C. (2002). Incubation and the costs of reproduction. Avian Incubation: Behaviour, Environment, and Evolution.

[6] Houston A.I., Székely T., McNamara J.M. (2005). Conflict between parents over care. Trends Ecol. Evol..

[7] Kokko H., Jennions M.D. (2008). Parental investment, sexual selection and sex ratios. J. Evol. Biol..

[8] Bulla M., Prüter H., Vitnerová H., Tijsen W., Sládeček M., Alves J.A., Gilg O., Kempenaers B. (2017). Flexible parental care: Uniparental incubation in biparentally incubating shorebirds. Sci. Rep..

[9] AlRashidi M., Kosztolányi A., Küpper C., Cuthill I.C., Javed S., Székely T. (2010). The influence of a hot environment on parental cooperation of a ground-nesting shorebird, the Kentish plover *Charadrius alexandrinus*. Front. Zool..

[10] AlRashidi M., Kosztolányi A., Shobrak M., Küpper C., Székely T. (2011). Parental cooperation in an extreme hot environment: Natural behaviour and experimental evidence. Anim. Behav..

[11] Hunter I., Clausen K.K., Bregnballe T. (2024). Nest attendance of the Kentish plover *Charadrius alexandrinus* in relation to temperature and incubation stage. Bird. Study.

[12] Huang C., Que P., Xu N., Yuan S., Zhang Z. (2026). Hourly analyses reveal environment-linked between-parent dynamics in a biparentally incubating shorebird. Avian Res..

[13] Boughdiri H., McDonald G.C., Székely T., Kosztolányi A. (2026). No evidence that nest cover shapes incubation schedules in a Mediterranean shorebird population. Ecol. Evol..

[14] Bulla M., Valcu M., Rutten A.L., Kempenaers B. (2014). Biparental incubation patterns in a high-Arctic breeding shorebird: How do pairs divide their duties?. Behav. Ecol..

[15] Bulla M., Valcu M., Dokter A.M., Dondua A.G., Kosztolányi A., Rutten A.L., Helm B., Sandercock B.K., Casler B., Ens B.J. (2016). Unexpected diversity in socially synchronized rhythms of shorebirds. Nature.

[16] Ekanayake K.B., Weston M.A., Nimmo D.G., Maguire G.S., Endler J.A., Küpper C. (2015). The bright incubate at night: Sexual dichromatism and adaptive incubation division in an open-nesting shorebird. Proc. R. Soc. B.

[17] Gochfeld M. (1978). Incubation behaviour in Common Terns: Influence of wind speed and direction on orientation of incubating adults. Anim. Behav..

[18] Lustick S., Battersby B., Kelty M. (1978). Behavioral thermoregulation: Orientation toward the sun in herring gulls. Science.

[19] Jennings M.C. (2010). Atlas of the Breeding Birds of Arabia.

[20] Maleko P.N., Billerman S.M., Pyle P., Billerman S.M. (2023). Black-winged Stilt (*Himantopus himantopus*), version 2.0. Birds of the World.

[21] Cuervo J.J. (2003). Parental roles and mating system in the Black-winged Stilt. Can. J. Zool..

[22] AlRashidi M., Shobrak M. (2025). Behavioural mechanisms employed by adult black-winged Stilts to mitigate heat stress in the hot environment of the eastern coast of Saudi Arabia. Avian Biol. Res..

[23] Key Biodiversity Areas Partnership Key Biodiversity Areas Factsheet: Dawmat al-Jandl Wetland. World Database of Key Biodiversity Areas. https://www.keybiodiversityareas.org/en/site/factsheet/8254.

[24] Green A.A. (1984). The avifauna of the Al Jawf, northwest Saudi Arabia. Sandgrouse.

[25] Pebesma E. (2018). Simple Features for R: Standardized Support for Spatial Vector Data. R J..

[26] Wickham H. (2016). ggplot2: Elegant Graphics for Data Analysis.

[27] Natural Earth Natural Earth: Free Vector and Raster Map Data. https://www.naturalearthdata.com/.

[28] OpenStreetMap Contributors OpenStreetMap [Map Data]. https://www.openstreetmap.org/copyright.

[29] Gorelick N., Hancher M., Dixon M., Ilyushchenko S., Thau D., Moore R. (2017). Google Earth Engine: Planetary-scale geospatial analysis for everyone. Remote Sens. Environ..

[30] Muñoz-Sabater J., Dutra E., Agustí-Panareda A., Albergel C., Arduini G., Balsamo G., Boussetta S., Choulga M., Harrigan S., Hersbach H. (2021). ERA5-Land: A state-of-the-art global reanalysis dataset for land applications. Earth Syst. Sci. Data.

[31] Hersbach H., Bell B., Berrisford P., Hirahara S., Horányi A., Muñoz-Sabater J., Nicolas J., Peubey C., Radu R., Schepers D. (2020). The ERA5 global reanalysis. Q. J. R. Meteorol. Soc..

[32] Alduchov O.A., Eskridge R.E. (1996). Improved Magnus form approximation of saturation vapor pressure. J. Appl. Meteorol..

[33] Thieurmel B., Elmarhraoui A. (2022). suncalc: Compute Sun Position, Sunlight Phases, Moon Position and Lunar Phase.

[34] R Core Team (2024). R: A Language and Environment for Statistical Computing.

[35] Brooks M.E., Kristensen K., van Benthem K.J., Magnusson A., Berg C.W., Nielsen A., Skaug H.J., Mächler M., Bolker B.M. (2017). glmmTMB balances speed and flexibility among packages for zero-inflated generalized linear mixed modeling. R J..

[36] Pinheiro J.C., Bates D.M. (2000). Mixed-Effects Models in S and S-PLUS.

[37] Manly B.F.J. (2007). Randomization, Bootstrap and Monte Carlo Methods in Biology.

[38] Efron B., Tibshirani R.J. (1993). An Introduction to the Bootstrap.

[39] Bolker B.M., Brooks M.E., Clark C.J., Geange S.W., Poulsen J.R., Stevens M.H.H., White J.-S.S. (2009). Generalized linear mixed models: A practical guide for ecology and evolution. Trends Ecol. Evol..

[40] Holm S. (1979). A Simple Sequentially Rejective Multiple Test Procedure. Scand. J. Stat..

[41] Lenth R.V., Piaskowski J. (2026). emmeans: Estimated Marginal Means, aka Least-Squares Means.

[42] McFadden D., Zarembka P. (1974). Conditional Logit Analysis of Qualitative Choice Behavior. Frontiers in Econometrics.

[43] Therneau T.M. (2024). A Package for Survival Analysis in R.

[44] Hartig F. (2024). DHARMa: Residual Diagnostics for Hierarchical (Multi-Level/Mixed) Regression Models.

[45] Sládeček M., Vozabulová E., Šálek M.E., Bulla M. (2019). Diversity of incubation rhythms in a facultatively uniparental shorebird—The Northern Lapwing. Sci. Rep..

[46] Kosztolányi A., Cuthill I.C., Székely T. (2009). Negotiation between parents over care: Reversible compensation during incubation. Behav. Ecol..

[47] Amat J.A., Masero J.A. (2004). How Kentish plovers, *Charadrius alexandrinus*, cope with heat stress during incubation. Behav. Ecol. Sociobiol..

[48] AlRashidi M. (2016). The challenge of coping in an extremely hot environment: A case study of the incubation of Lesser Crested Terns (*Thalasseus bengalensis*). Waterbirds.

[49] AlRashidi M. (2025). Breeding ecology and incubation behaviours of Little Tern *Sternula albifrons* in the Sabkhat Al-Fasl Lagoons, Saudi Arabia. Mar. Ornithol..

[50] AlRashidi M., Alatawi A.S., Shobrak M., Abdelgadir M. (2025). Incubation behavior of the Western Reef Heron (*Egretta gularis*) in Eastern Saudi Arabia: Adaptations to extreme thermal conditions. Life.

[51] AlRashidi M., Rice R., Almalki A., Alzoubi M., Shobrak M. (2025). Parental care of the Cream-coloured Courser (*Cursorius cursor*) in the harsh environment of northern Saudi Arabia. Zool. Middle East.

[52] Zbyryt A., Jankowiak Ł., Jerzak L., Tryjanowski P. (2022). Head and body orientation of the White Stork *Ciconia ciconia* during incubation: Effect of wind, apex predators and power lines. J. Ornithol..

[53] Ward D. (1990). Incubation Temperatures and Behavior of Crowned, Black-Winged, and Lesser Black-Winged Plovers. Auk.

[54] Cestari C., de Melo C. (2022). Shorebirds adjust resting orientation in response to solar radiation and wind speed. Austral Ecol..

[55] Downs C.T., Ward D. (1997). Does shading behavior of incubating shorebirds in hot environments cool the eggs or the adults?. Auk.

