# Supplementary material for: Sex-Specific Incubation and Body Orientation in the Black-Winged Stilt (Himantopus himantopus): Diel Patterns and Environmental Associations
Source: Animals (Basel). 2026 Nov 2;16(21):3467. doi: 10.3390/ani16213467 (PMC13761600; doi:10.3390/ani16213467)
Supplement: Supplementary file 1 [file animals-16-03467-s001.zip › animals-4609035-supplementary.pdf]

1. Supplementary Tables S1-S7 and Figures S1-S3

Black-winged Stilt: sex-specific incubation and orientation

Table S1. Nest-level incubation attendance and sex-specific contributions during 24-h monitoring periods.

Nest ID	Study site	Female (min)	Male (min)	No incubation (min)	Total incubation (min)	Incubation (%)	Male share (%)
BWS01	Dumat Al-Jandal	585	681	174	1,266	87.92%	53.79%
BWS015	Dumat Al-Jandal	793	579	68	1,372	95.28%	42.20%
BWS016	Dumat Al-Jandal	666	609	165	1,275	88.54%	47.76%
BWS018	Dumat Al-Jandal	528	630	282	1,158	80.42%	54.40%
BWS02_1	Dumat Al-Jandal	747	568	125	1,315	91.32%	43.19%
BWS406	Dumat Al-Jandal	553	692	195	1,245	86.46%	55.58%
BWS_259	Sabkhat Al-Fasl	511	873	56	1,384	96.11%	63.08%
BWS_614	Sabkhat Al-Fasl	582	699	159	1,281	88.96%	54.57%
BWS_618	Sabkhat Al-Fasl	648	752	40	1,400	97.22%	53.71%
BWS_627	Sabkhat Al-Fasl	786	614	40	1,400	97.22%	43.86%
BWS304_1	Sabkhat Al-Fasl	696	708	36	1,404	97.50%	50.43%
BWS406_1	Sabkhat Al-Fasl	766	654	20	1,420	98.61%	46.06%

Note. 1,440 recorded minutes per nest. Nest-mean male share = 50.72%; pooled male share = 50.62%; different weighting.

Table S2. Male incubation share for each two-hour period, summarized across nests.

Two-hour period	N nests	Mean male share (%)	SD (pp)	SE (pp)
00–02	12	56.6%	32.5	9.4
02–04	12	57.2%	40.4	11.7
04–06	12	62.6%	21.5	6.2
06–08	12	42.0%	20.8	6.0
08–10	12	34.4%	26.7	7.7
10–12	12	49.1%	21.0	6.1
12–14	12	53.6%	12.3	3.6
14–16	12	53.7%	15.2	4.4
16–18	12	56.8%	32.5	9.4
18–20	12	46.3%	35.4	10.2
20–22	12	71.2%	31.6	9.1
22–24	12	31.9%	31.6	9.1

Note. Male and female shares are complementary; raw period counts appear in the analysis archive. SD and SE are expressed in percentage points (pp).

Table S3. Adjusted solar-relative orientation probabilities by sex and daylight clock period.

Two-hour period	Sex	Adjusted probability	Model 95% CI
04–06	Female	50.3%	31.3% to 69.2%
06–08	Female	49.9%	33.1% to 66.7%
08–10	Female	49.4%	32.3% to 66.5%
10–12	Female	68.2%	51.6% to 81.2%
12–14	Female	82.3%	68.8% to 90.7%
14–16	Female	57.0%	40.1% to 72.5%
16–18	Female	46.2%	29.3% to 64.1%
18–20	Female	38.8%	22.3% to 58.4%
04–06	Male	43.7%	25.7% to 63.4%
06–08	Male	43.2%	27.2% to 60.9%
08–10	Male	42.7%	26.4% to 60.8%
10–12	Male	62.1%	45.0% to 76.7%
12–14	Male	78.0%	63.3% to 88.0%
14–16	Male	50.4%	33.9% to 66.8%
16–18	Male	39.7%	24.3% to 57.5%
18–20	Male	32.7%	17.8% to 52.2%

Note. Probabilities and confidence intervals are model-based; compass-preference confounding cannot be excluded.

Table S4. Representative sex-specific wind-facing probabilities at selected observed standardized wind speeds.

Sex	Wind speed (SD)	Adjusted probability	Model 95% CI
Female	-1.04	29.0%	18.1% to 42.9%
Female	0.03	52.1%	40.4% to 63.5%
Female	0.98	72.4%	58.7% to 82.9%
Male	-1.04	46.3%	32.2% to 61.0%
Male	0.03	62.0%	50.4% to 72.3%
Male	0.98	74.3%	61.2% to 84.1%

Note. Selected grid points are descriptive predictions, not separate significance tests.

Table S5. DHARMa dispersion and residual diagnostics for the primary sex-comparison models and two-hour-period model checks.

Model	Simulation	N simulations	Dispersion ratio	Dispersion P	Uniformity P	Outlier P	Source
Incubation period	Unconditional	1000	0.682	<0.001	0.300	—	V7.0.7
Incubation period	Conditional	1000	0.682	<0.001	0.385	—	V7.0.7
Incubation environment	Unconditional	1000	0.684	<0.001	0.412	—	V7.0.7
Incubation environment	Conditional	1000	0.683	<0.001	0.350	—	V7.0.7
Solar sex	Unconditional	1000	0.893	0.252	0.931	—	V7.0.7
Solar sex	Conditional	1000	0.965	0.682	0.661	—	V7.0.7
Wind sex	Unconditional	1000	0.860	0.036	0.951	—	V7.0.7
Wind sex	Conditional	1000	0.919	0.212	0.078	—	V7.0.7
Reduced null	Unconditional	1000	0.691	<0.001	0.001	1.000	V7.0.8b
Reduced null	Conditional	1000	0.692	<0.001	<0.001	1.000	V7.0.8b
Full period	Unconditional	1000	0.683	<0.001	0.369	1.000	V7.0.8b
Full period	Conditional	1000	0.681	<0.001	0.350	1.000	V7.0.8b
Speed_ge_2	Unconditional	1000	0.881	0.094	0.883	1.000	V7.0.10b
Speed_ge_2	Conditional	1000	0.918	0.192	0.171	1.000	V7.0.10b
Steadiness_ge_0.90	Unconditional	1000	0.869	0.070	0.969	1.000	V7.0.10b
Steadiness_ge_0.90	Conditional	1000	0.925	0.260	0.274	1.000	V7.0.10b
Both_filters	Unconditional	1000	0.874	0.072	0.973	1.000	V7.0.10b
Both_filters	Conditional	1000	0.912	0.162	0.175	1.000	V7.0.10b

Note. Unconditional and conditional-random-effect simulations are separate. Residual underdispersion remains an unresolved model limitation; cyclic-shift sensitivity is separate.

Table S5 (continued). Wind-filter sensitivity of sex-related likelihood-ratio tests (V7.0.10).

Wind-data filter	N nests	N windows	Minutes	Joint sex P	Sex x wind P
Full	12	8	15,920	0.015	0.071
Speed_ge_2	12	8	14,015	0.031	0.070
Steadiness_ge_0.90	12	8	15,209	0.042	0.132
Both_filters	12	8	13,898	0.031	0.067

Note. Asymptotic likelihood-ratio tests of sex-related terms, not equivalence tests. Shared meteorological monitoring windows limit inference; wind filters are exploratory, not biological thresholds.

Table S6. Compass-direction distributions, within-attender direction changes and orientation episodes by nest and sex.

Nest ID	Sex	N (%)	E (%)	S (%)	W (%)	Changes	Within-sex transitions	Orientation episodes	Camera position
BWS_259	Female	53.0%	29.0%	6.5%	11.5%	17	481	47	North
BWS_259	Male	37.8%	25.4%	8.0%	28.8%	52	836	89	North
BWS_614	Female	11.9%	17.0%	41.2%	29.9%	58	548	92	East
BWS_614	Male	23.3%	16.0%	28.0%	32.6%	52	656	95	East
BWS_618	Female	22.7%	8.3%	39.7%	29.3%	17	609	56	South
BWS_618	Male	31.5%	30.5%	5.1%	33.0%	30	710	72	South
BWS_627	Female	13.0%	29.8%	34.7%	22.5%	30	747	69	South
BWS_627	Male	9.6%	36.8%	11.2%	42.3%	17	565	66	South
BWS01	Female	31.5%	33.7%	12.3%	22.6%	35	559	61	West
BWS01	Male	32.0%	39.6%	1.3%	27.0%	9	663	27	West
BWS015	Female	50.6%	20.9%	12.4%	16.1%	25	764	54	North
BWS015	Male	55.8%	31.3%	6.4%	6.6%	18	558	39	North
BWS016	Female	56.9%	24.0%	2.3%	16.8%	26	648	44	West
BWS016	Male	60.1%	10.8%	15.3%	13.8%	18	584	43	West
BWS018	Female	46.4%	45.6%	3.8%	4.2%	9	506	31	West
BWS018	Male	59.4%	27.6%	6.5%	6.5%	25	613	42	West
BWS02_1	Female	12.2%	2.7%	17.5%	67.6%	14	723	38	North
BWS02_1	Male	50.5%	21.0%	2.5%	26.1%	32	547	53	North
BWS304_1	Female	27.7%	26.9%	3.7%	41.7%	38	656	78	North
BWS304_1	Male	13.6%	25.1%	27.3%	34.0%	55	673	90	North
BWS406	Female	35.1%	21.7%	0.9%	42.3%	9	529	33	South
BWS406	Male	34.5%	33.2%	3.3%	28.9%	15	664	43	South
BWS406_1	Female	28.1%	24.9%	24.0%	23.0%	46	739	73	South
BWS406_1	Male	19.4%	11.0%	9.6%	59.9%	19	634	39	South

Note. N/E/S/W refer to rescored dirc2 incubated minutes. Episodes end on a sex/direction change or data gap; total 1374. Camera POSITION varies by nest (N/E/S/W; field report); exact numerical camera azimuths were not measured.

Table S7. Episode-onset and conditional-choice sensitivity analyses of sex differences in orientation.

Panel	Model / test	Sex comparison	Estimate	95% CI	Independent unit (n)	Bootstrap fits / exact P
A. Conditional choice	Solar	Male x solar-away alignment	-0.109	-0.212 to 0.001	Nest (12)	999/999 accepted
A. Conditional choice	Wind	Male x wind-facing alignment	0.088	-0.112 to 0.227	Monitoring window (8)	999/999 accepted
A. Conditional choice	Wind	Male x wind-facing x wind speed	-0.179	-0.337 to -0.008	Monitoring window (8)	999/999 accepted
A. Conditional choice	Joint angle	Male x solar alignment	-0.173	-0.344 to -0.047	Monitoring window (8)	499/499 accepted
A. Conditional choice	Joint angle	Male x wind-facing alignment	0.339	0.070 to 0.623	Monitoring window (8)	499/499 accepted
A. Conditional choice	Joint angle	Male x wind-facing x wind speed	-0.145	-0.345 to 0.005	Monitoring window (8)	499/499 accepted
A. Conditional choice	Joint horizontal	Male x solar alignment	-0.272	-0.651 to -0.007	Monitoring window (8)	495/499 accepted
A. Conditional choice	Joint horizontal	Male x wind-facing alignment	0.364	0.021 to 0.836	Monitoring window (8)	495/499 accepted
A. Conditional choice	Joint horizontal	Male x wind-facing x wind speed	-0.135	-0.432 to -0.016	Monitoring window (8)	495/499 accepted
B. Episode-onset	Solar excess alignment	Male minus female; compass-adjusted	-0.054	-0.127 to 0.020	Nest (12)	Exact P = 0.218
B. Episode-onset	Wind excess alignment	Male minus female; compass-adjusted	-0.000	-0.048 to 0.036	Monitoring window (8)	Exact P = 1.000
B. Episode-onset	Wind-speed slope	Male minus female; compass-adjusted	-0.107	-0.227 to -0.003	Monitoring window (8)	Exact P = 0.094

Note. Exploratory sensitivity analyses of male-female orientation contrasts, not primary models. Panel A values are conditional-choice log-utility coefficients with percentile 95% cluster-bootstrap CIs (Solar: resampled nests, n=12; other models: resampled monitoring windows, n=8); accepted/requested fits are shown, not hypothesis-test P values. Each model conditions on nest-by-sex cardinal-direction baseline preferences. Panel B values are male-minus-female differences in excess compass-adjusted alignment (or wind-speed slope); percentile CIs and exact paired sign-flip P values are based on 12 nests or 8 monitoring windows, as stated. Panel A and B estimates are on different scales and must not be directly compared. Episode onset is approximated by the first photographed minute of each run (1374 episodes, recomputed from verified dirc2); consecutive episodes may remain temporally related. CI overlap with zero does not demonstrate sex equivalence, and these analyses do not rank solar versus wind effects.

2. Supplementary Figures

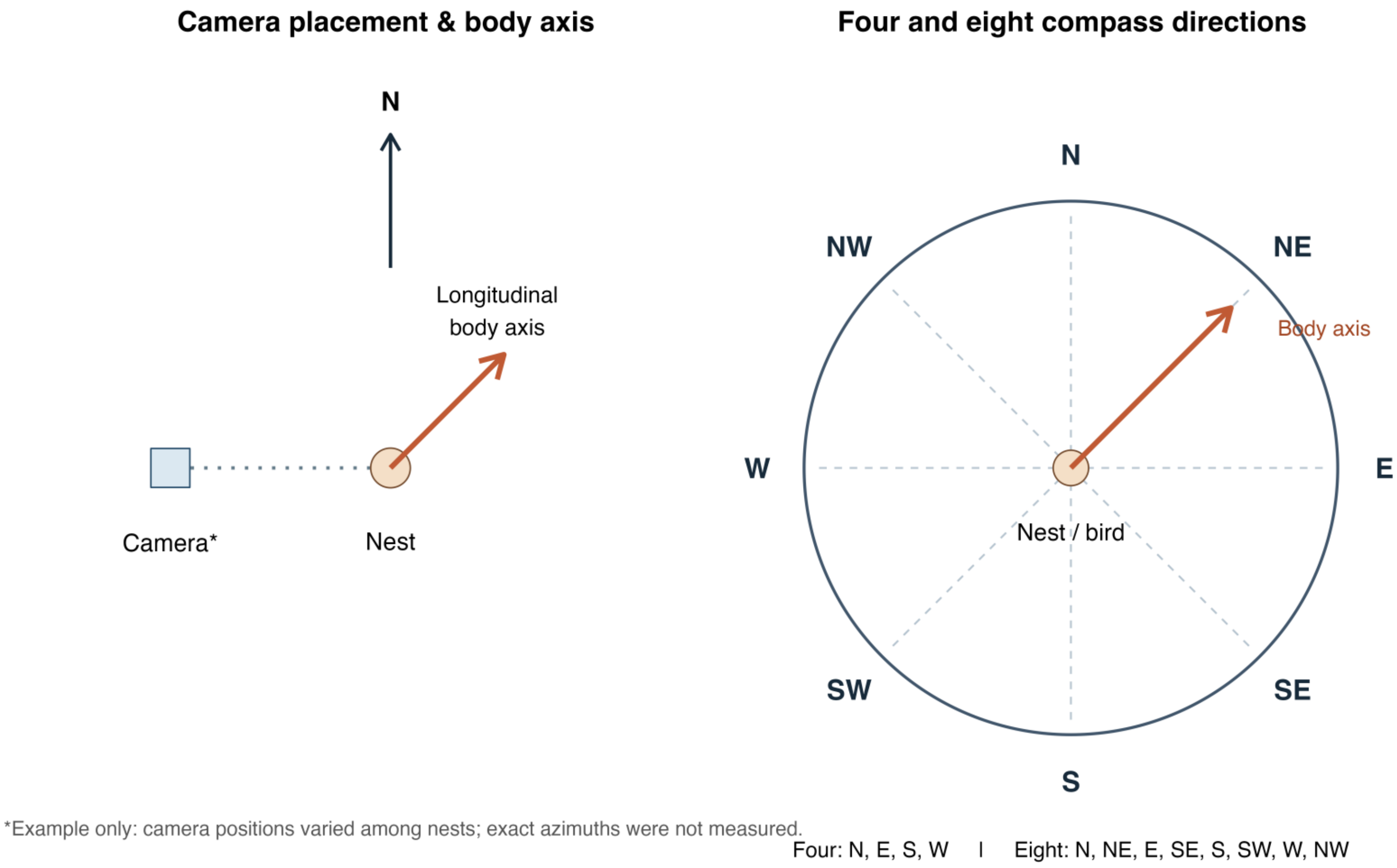

Figure S1. Schematic of nest-camera placement, the bird's longitudinal body axis, and four-/eight-direction compass classification. The example camera position and body orientation are illustrative, not actual field azimuth measurements. Cameras occupied different positions among nests; precise camera azimuths were not recorded.

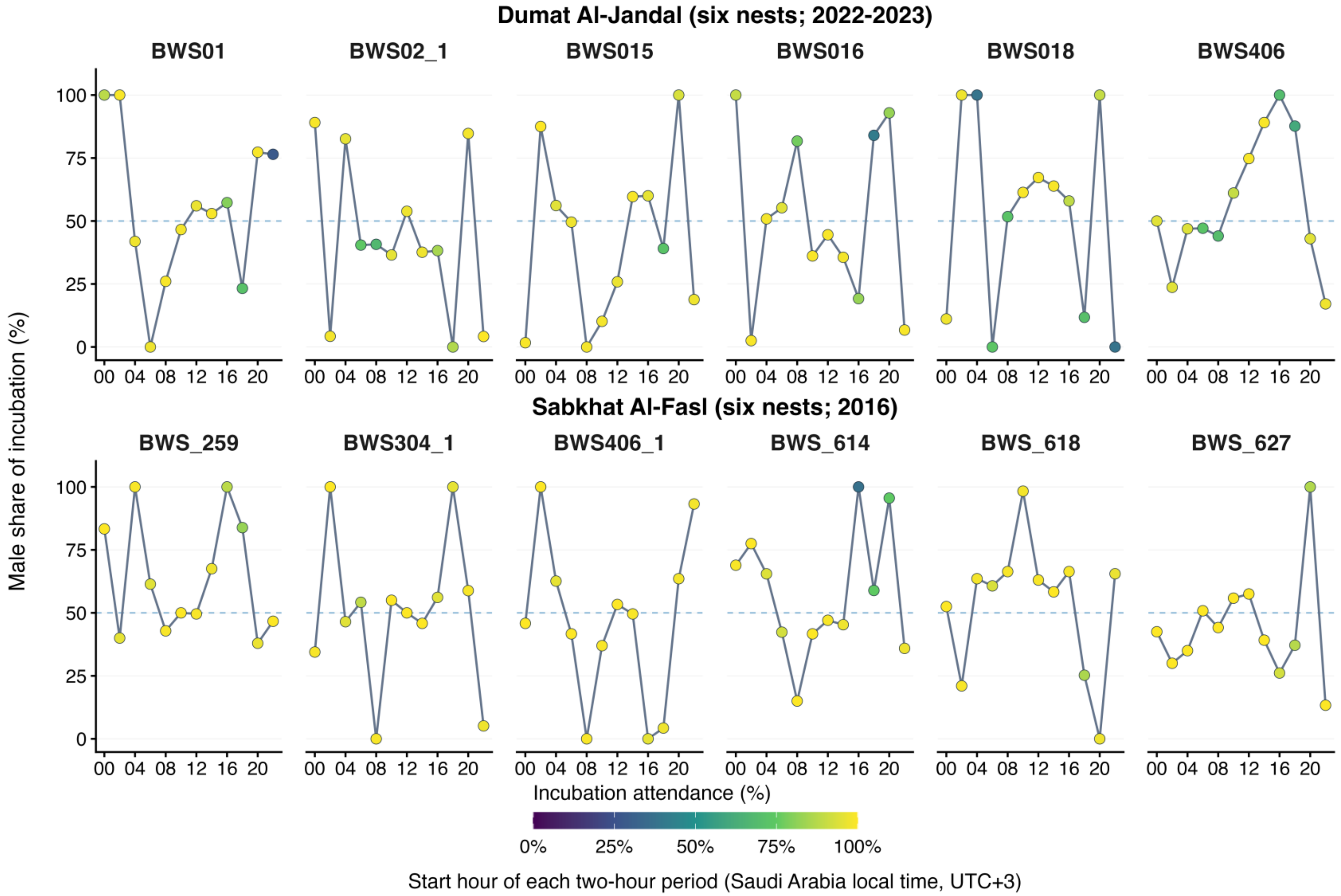

Figure S2. Nest-specific variation in the proportion of attended incubation minutes contributed by males across 12 two-hour clock periods. The upper row contains six Dumat Al-Jandal nests (2022–2023); the lower row contains six Sabkhat Al-Fasl nests (2016). Point colour represents incubation attendance (%), defined as the percentage of each 120-min period during which either parent incubated; the vertical axis shows the male percentage of the minutes attended by either parent; the horizontal dashed line shows 50%. Connected lines are descriptive only, not fitted trends or independent measurements. Each nest was recorded during one 24-h interval; Saudi Arabia local clock time (UTC+3).

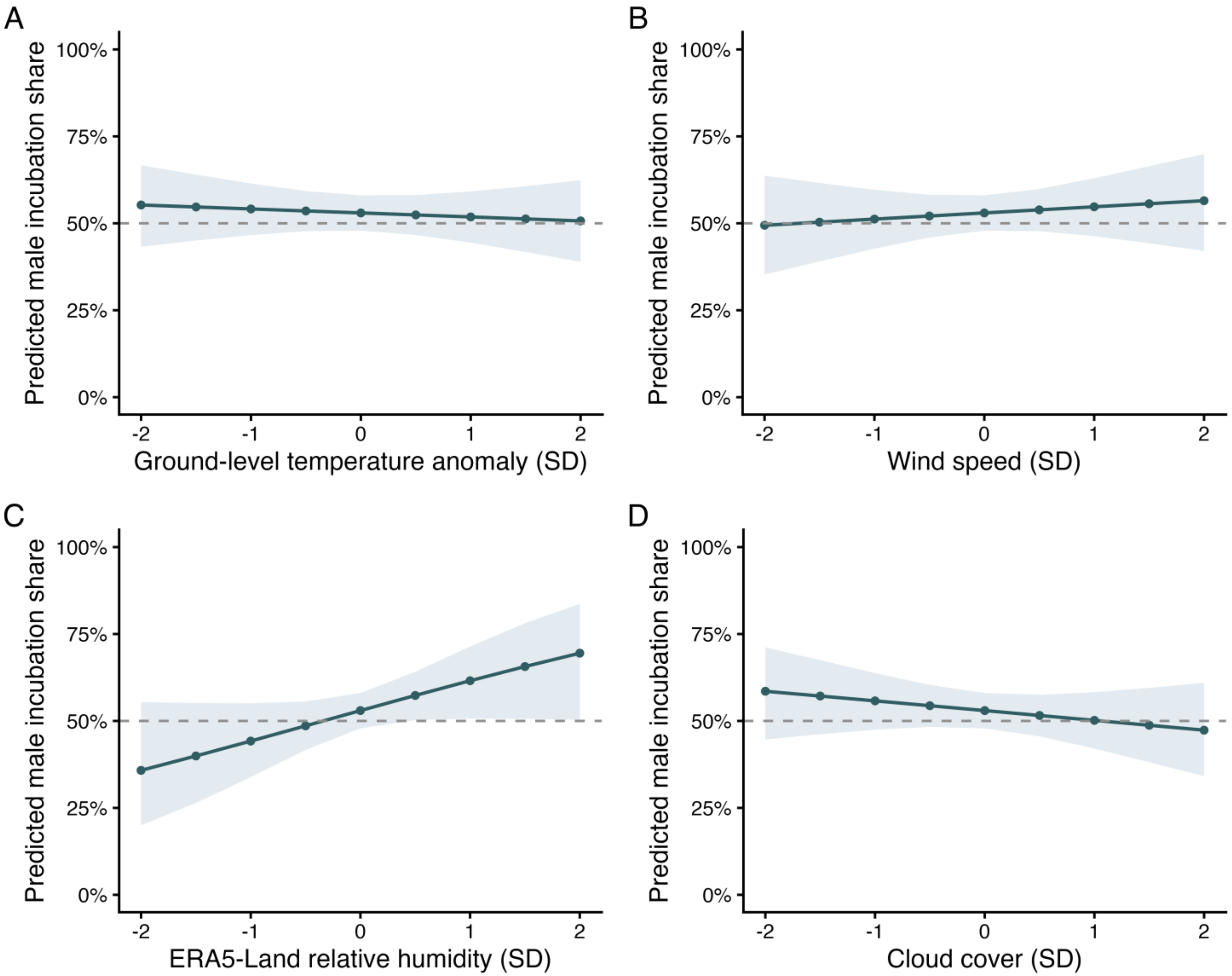

Figure S3. Adjusted associations between male incubation share and (A) ground-level temperature anomaly, (B) wind speed, (C) ERA5-Land relative humidity, and (D) cloud cover. Lines and shaded bands are model predictions and nominal 95% model confidence intervals. Environmental inference was exploratory; none of the four associations was supported after Holm correction.
